# Call to action: British and Irish hypertension society position statement on blood pressure treatment thresholds and targets

**DOI:** 10.1038/s41371-025-01055-z

**Published:** 2025-07-23

**Authors:** Luca Faconti, Nayanatara Tantirige, Neil R. Poulter, Jacob George, Vikas Kapil, Ajay Gupta, Pauline A. Swift, Anthony Heagerty, Eduard Shantsila, Sarah Partridge, Ian B. Wilkinson

**Affiliations:** 1 King’s College London British Heart Foundation Centre, Department of Clinical Pharmacology, 4th Floor, North Wing, St. Thomas’ Hospital, Westminster Bridge, London, SE1 7EH UK; 2 Division of Experimental Medicine, University of Cambridge, Addenbrooke’s Hospital, Cambridge, CB20QQ UK; 3 Imperial Clinical Trials Unit, School of Public Health, Imperial College London, London, W12 7RH UK; 4 Division of Molecular & Clinical Medicine, School of Medicine, Ninewells Hospital & Medical School, University of Dundee, Dundee, DD1 9SY UK; 5 William Harvey Research Institute, Centre for Cardiovascular Medicine and Devices, Queen Mary University London, London, EC1M 6BQ UK; 6 William Harvey Research Institute, Clinical Pharmacology and Precision Medicine, Queen Mary University of London, London, EC1M 6BQ UK; 7 Renal Department, Epsom & St Helier University Hospitals NHS Trust, London, SM5 1AA UK; 8 Division of Cardiovascular Sciences, University of Manchester, Manchester, M13 9NT UK; 9 Department of Primary Care and Mental Health, University of Liverpool, Liverpool, L69 7ZX UK; 10 Brighton and Sussex Medical School, University of Sussex, Brighton, BN1 9PH UK

**Keywords:** Hypertension, Diagnosis, Preventive medicine

## Abstract

In this position statement the British and Irish Hypertension Society (BIHS) present a review of the current evidence for blood pressure (BP) treatment thresholds and targets. The BIHS recommend initiating pharmacological antihypertensive therapy, irrespective of cardiovascular disease risk, following a confirmed diagnosis of hypertension (sustained out-of-office BP ≥ 135/85 mmHg despite diet and lifestyle advice). The BIHS recommend an on-treatment BP target < 130/80 mmHg or as low as reasonably achievable without causing unacceptable side-effects, within 6-months of initiating treatment, for all adults. Possible subgroups to whom this may not apply are those who are frail and/or have limited life expectancy where higher targets may be appropriate based on clinical judgement and the individuals’ tolerance to treatment. The BIHS believe that this simple 2-step approach will facilitate practitioners deliver evidence-based best practice, discourage therapeutic inertia around BP lowering and improve heath outcomes for all adults living with high BP.

## Statement

High blood pressure (BP) is the leading modifiable risk factor for premature morbidity and mortality from cardiovascular disease (CVD) worldwide, and significantly contributes to chronic kidney disease, heart failure and dementia [1, 2].

Epidemiological studies demonstrate a continuous positive association between BP and major adverse cardiovascular events (MACE) [3–5]. Observational data are supported by randomized clinical trials, demonstrating the benefits of antihypertensive drug treatment in reducing MACE [6, 7]. The Blood Pressure Lowering Treatment Trialists’ Collaboration reported that the reduction in MACE was proportional to the magnitude of the fall in SBP down to at least 120 mmHg in those aged < 75 years, with and without prior cardiovascular disease [6, 7]. Overall, MACE was reduced by 10% per 5 mmHg reduction in Systolic BP (SBP), with a greater effect in younger (age <55 years: HR 0·82 [95% CI: 0·76–0·88]) compared with older individuals (age 65–74 years: HR 0·91 [95%CI: 0·87–0·96]) [6, 7].

Over the last 15 years, several large outcome trials have been conducted to specifically compare aiming for an intensive BP treatment target (e.g. SBP < 120 mmHg) with a less intensive target (e.g. SBP < 140 mmHg) among men and women, with and without pre-existing CVD and diabetes [8–14]. These trials consistently demonstrate that aiming for an SBP treatment target <120 mmHg resulted in a greater reduction in MACE compared with an SBP target < 140 mmHg, with no increase in all-cause mortality [Table 1] [8–14]. There was no overall significant difference in reported serious adverse events between treatment groups [Table 1] [8–14].

**Table 1:** Summary of trials comparing intensive vs standard systolic blood pressure targets.

	ACCORD, 2010 [8]	SPS3, 2013 [9]	SPRINT, 2015/2021 [10]	RESPECT, 2019 [11]	STEP, 2021 [12]	ESPRIT, 2024 [13]	BPROAD, 2024 [14]
No. of Participants	4733	3020	9361	1263	8511	11,255	12,821
Inclusion Criteria	T2DM, elevated SBP, increased risk of CVD, ≥ 40 years old.	Recent lacunar stroke, ≥ 30 years old, normotensive or hypertensive, without surgically amenable ipsilateral carotid artery stenosis or high-risk cardioembolic sources.	Elevated SBP, increased risk of CVD, ≥ 50 years old. Diabetes and prior stroke excluded.	Stroke, elevated SBP, ≥ 50 years old.	Elevated SBP, 60–80 years old, prior stroke excluded.	Elevated SBP, increased risk of CVD, ≥ 50 years old.	T2DM, elevated SBP, increased risk of CVD and ≥50 years old.
Mean Age at Baseline (years)	62.2	63	67.9	67.2	66.2	64.6	63.8
Mean BP at Baseline (mmHg)	139.2/76.0 mmHg	143/78.5 mmHg	139.7/78.2 mmHg	145.4/83.6 mmHg	146/82.5 mmHg	146.9/82.9 mmHg	140/76 mmHg
BP Measurement	Attended office automatic BP measurement	Attended office automatic BP measurement	Unattended office automated BP measurement	Attended office automatic BP measurement	Attended office automatic BP measurement	Attended office automatic BP measurement	Attended office automatic BP measurements (home BP monitoring during COVID-19)
Target BP: Intervention vs Control Group	SBP < 120 mmHg vs <140 mmHg	SBP < 130 mmHg vs 130–149 mmHg	SBP < 120 mmHg vs <140 mmHg	SBP < 120 mmHg vs <140 mmHg	SBP 110- < 130 mmHg vs 130- < 150 mmHg	SBP < 120 mmHg vs <140 mmHg	SBP < 120 mmHg vs <140 mmHg
Mean Achieved Systolic BP (intervention vs control group)	119.3 mmHg vs 133.5 mmHg (at 1 year)	127 mmHg vs 138 mmHg (at 1 year)	121.4 mmHg vs 136.2 mmHg (at 1 year)	126.7 mmHg vs 133.2 mmHg (mean throughout follow-up)	127.5 mmHg vs 135.3 mm Hg (at 1 year)	119.1 mmHg vs 134.8 mmHg (mean throughout follow-up)	SBP 121.6 vs 133.2 (at 1 year)
Primary Outcome – Major Cardiovascular Adverse Events (intervention vs control group)	208 vs 237 patients HR 0.88 (95% CI 0.73–1.06)	125 vs 152 patients HR 0.81 (95% CI 0.64–1.03) Primary outcome was recurrence of stroke.	264 vs 354 patients HR 0.73 (95% CI 0.63–0.86)	46 vs 59 patients HR 0.76 (95% CI 0.52–1.12) Primary outcome was recurrence of stroke. Trial terminated early.	147 vs 196 patients HR 0.74 (95% CI 0.60–0.92)	547 vs 623 patients HR 0.88 (95% CI 0.78–0.99)	393 vs 492 patients HR 0.79 (95% CI 0.69–0.90)
All-Cause Mortality (Intervention vs Control Group)	1.28% vs 1.19% per year HR 1.07 (95% CI 0.85–1.35)	1.80% vs 1.74% per patient-year HR 1.03 (95% CI 0.79–1.35)	1.06% vs 1.41% per year HR 0.75 (95%CI 0.61–0.92)	30 vs 37 patients HR 0.80 (95% CI 0.49–1.29)	67 vs 64 patients HR 1.11 (95%CI 0.78–1.56)	2.8% vs 3.6% HR 0·79 (95%CI 0·64–0·97)	0.69 vs 0.73 events/ 100 person-years HR 0.95 (95% CI 0.77–1.17)
Serious Adverse Events (Intervention vs Control Group)	77 (3.3%) vs 30 (1.3%) HR not calculated	23 (0.40% per patient-year) vs 15 (0.26% per patient-year) HR 1.53 (95% CI 0.80–2.93)	1799 (38.5%) vs 1742 (37.2%) HR 1.04 (95% CI 0.97–1.11)	179 (28.3%) vs 178 (28.3%) HR not calculated	21 (0.5%) vs 21 (0.5%) HR not calculated	2366 (42.1%) vs 2378 (42.2%) HR 1.01 (95% CI 0.95–1.07)	2340 (36.5%) vs 2328 (36.3%) HR 1.0 (95% CI 0.94–1.06)

*BP* blood pressure, *CVD* cardiovascular disease, *HR* hazard ratio, *SBP* systolic blood pressure, *T2DM* type 2 diabetes mellitus.

**Fig. 1:**
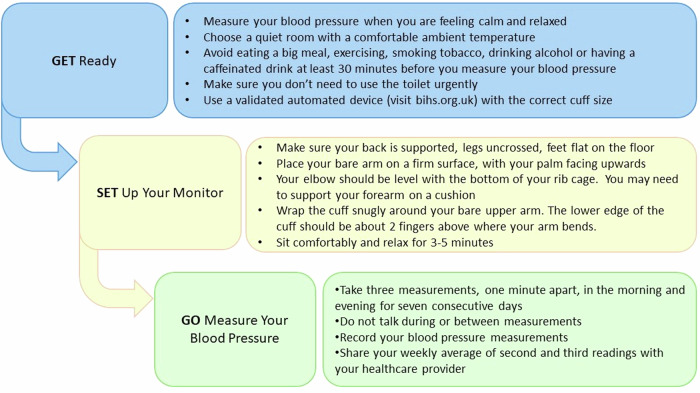
BIHS Guide to Home Blood Pressure Measurement.

Results were consistent across these trials whether BP was measured by attended or unattended office readings or home BP measurements (HBPM) [8–14]. This aligns with a UK study by McManus and colleagues which demonstrated that at lower levels of BP the difference between office and HBPM was small [15]. Based on this evidence, the British and Irish Hypertension Society (BIHS) recommend that BP targets remain the same whether BP is measured in the office, by 7-day average HBPM [Fig. 1] or day-time average ambulatory BP measurement (ABPM).

The current National Institute of Clinical Excellence (NICE) Adult Hypertension Guideline NG136, recommended antihypertensive treatment to be initiated based on a combination of BP-level and 10-year CVD risk [16]. This approach targets treatment to those at highest short-term absolute risk. In light of the continuing excess of premature morbidity and mortality from BP-related diseases [1, 2], the BIHS questions the ethics of waiting until sufficient irreversible damage has been done to meet an arbitrary risk threshold, before cheap, safe and effective antihypertensive treatment (both lifestyle and pharmacological) is initiated.

Current NICE BP treatment targets for people aged under 80 years are <140/90 mmHg, with <130/80 mmHg only recommended in specific clinical situations (e.g. some patients with diabetes and chronic kidney disease) [16]. The BIHS believes there is now sufficient new evidence to recommend one consistent BP target of <130/80 mmHg or as low as reasonably achievable (ALARA) without causing unacceptable side-effects within 6-months of initiating treatment, among **all** adults with a confirmed diagnosis of hypertension. Possible subgroups to whom this may not apply are those who are frail and/or have limited life expectancy where higher targets may be appropriate based on clinical judgement and the individuals’ tolerance to treatment.

Thus, based on current evidence, the BIHS calls for NICE to update its guidance in NG136 “*Hypertension in adults: diagnosis and management*” [16] in line with current recommendations from Europe and the United States of America [17–20] as follows:

### BP threshold for treatment

Following a confirmed diagnosis of hypertension (sustained BP ≥ 135/85 mmHg based on 7-day average HBPM or day-time average ABPM despite diet and lifestyle advice) starting BP-lowering treatment with pharmacological therapy on top of diet and lifestyle interventions is recommended irrespective of CVD risk. We recommend the BIHS algorithm in “*Adult Hypertension Referral Pathway and Therapeutic Management: British and Irish Hypertension Society Position Statement*” for a titration protocol and exemplar therapeutic options within the NICE treatment pathway [21].

### On-treatment BP target

On treatment BP target < 130/80 mmHg (measured by 7-day average HBPM **or** day-time average ABPM **or** office BP*) or ALARA without causing unacceptable side-effects, and within 6-months of initiating treatment, for **all** adults. Possible subgroups to whom this may not apply are those who are frail and/or have limited life expectancy where higher targets may be appropriate based on clinical judgement and the individuals’ tolerance to treatment.

^*^Among people with diagnosed white-coat hypertension out-of-office measurements should always be used to evaluate treatment.

The BIHS believe that this simple 2-step approach will facilitate practitioners deliver evidence-based best practice, discourage therapeutic inertia around BP lowering and improve health outcomes for all adults living with high BP.

## Data Availability

All data generated and analysed for the development of this statement are included in this published article.
